# Intergenerational educational trajectories and inequalities in longevity: A population-based study of adults born before 1965 in 14 European countries

**DOI:** 10.1016/j.ssmph.2023.101367

**Published:** 2023-02-22

**Authors:** Cornelia Wagner, Stéphane Cullati, Stefan Sieber, Tim Huijts, Arnaud Chiolero, Cristian Carmeli

**Affiliations:** a Population Health Laboratory (#PopHealthLab), University of Fribourg, Switzerland; b Department of Readaptation and Geriatrics, University of Geneva, Switzerland; c LIVES Centre, Swiss Centre of Expertise in Life Course Research, University of Lausanne, Switzerland; d Research Centre for Education and the Labour Market (ROA), Maastricht University, the Netherlands; e Centre for Global Health Inequalities Research (CHAIN), Norwegian University for Science and Technology, Norway; f Institute of Primary Health Care (BIHAM), University of Bern, Switzerland; g School of Population and Global Health, McGill University, Canada

**Keywords:** All-cause mortality, Intergenerational educational trajectories, Health inequality, Life course, Longevity

## Abstract

**Background:**

While educational gradients in longevity have been observed consistently in adult Europeans, these inequalities have been understudied within the context of family- and country-level influences. We utilized population-based multi-generational multi-country data to assess the role (1) of parental and individual education in shaping intergenerational inequalities in longevity, and (2) of country-level social net expenditure in mitigating these inequalities.

**Methods:**

We analyzed data from 52,271 adults born before 1965 who participated in the Survey of Health, Ageing and Retirement in Europe, comprising 14 countries. Mortality from all causes (outcome) was ascertained between 2013 and 2020. Educational trajectories (exposure) were High-High (reference), Low-High, High-Low, and Low-Low, corresponding to the sequence of parental-individual educational attainment. We quantified inequalities as years of life lost (YLL) between the ages of 50 and 90 estimated via differences in the area under standardized survival curves. We assessed the association between country-level social net expenditure and YLL via meta-regression.

**Results:**

Inequalities in longevity due to educational trajectories were associated with low individual education regardless of parental education. Compared to High-High, having High-Low and Low-Low led to 2.2 (95% confidence intervals: 1.0 to 3.5) and 2.9 (2.2 to 3.6) YLL, while YLL for Low-High were 0.4 (−0.2 to 0.9). A 1% increase in social net expenditure led to an increase of 0.01 (−0.3 to 0.3) YLL for Low-High, 0.007 (−0.1 to 0.2) YLL for High-Low, and a decrease of 0.02 (−0.1 to 0.2) YLL for Low-Low.

**Conclusion:**

In European countries, individual education could be the main driver of inequalities in longevity for adults older than 50 years of age and born before 1965. Further, higher social expenditure is not associated with smaller educational inequalities in longevity.

## Highlights

• Educational gradients in longevity were tracked across generations among older adults from 14 European countries.
• Shortened longevity was associated with low individual education regardless of parental education.
• Higher social expenditure was not associated with smaller educational gradients in longevity.

## 1 Introduction

Educational gradients in longevity have been observed consistently in adult Europeans (Mackenbach et al., 2019), whereby a higher education is associated with a longer life expectancy. According to the fundamental cause theory (Masters et al., 2015), higher educational attainment grants access to resources such as social connections, higher income, higher labor market returns, or health-related knowledge, which all serve to improve health outcomes and eventually increase longevity. The putative causal effect of education has found empirical evidence via quasi-experiments in populations from the U.S., Sweden, and the United Kingdom (Davies et al., 2018; Lager & Torssander, 2012; Lleras-Muney, 2005). However, the extent to which educational attainment affects longevity may depend on contextual factors. Key contextual influences that are worth being studied are related to the familial environment and country of residence (Bambra et al., 2010).

The familial environment may contribute to the amount of cultural and social capital a person has access to, and eventually to social inequalities in offspring health. This can be explained via various pathways. Taking parental education as a proxy of the family/household socioeconomic status, one pathway is via differential access to material and social resources within society (Galobardes et al., 2007). Another pathway is via socialization of individuals into health behaviors typical of their social surroundings (Schuck & Steiber, 2018). Additionally, parental education is also an important cause of offspring education (Conley et al., 2015). Overall, parental education may set the stage for health inequalities that persist throughout the offspring life course, from childhood until death. What remains less clear is the interplay between parental and individual education. More specifically, which educational exposure – parental or individual – is the main driver of putative intergenerational inequalities in longevity?

Social theories predict different possible answers: cumulative advantage theory indicates that both parental and individual education drive inequalities (Willson et al., 2007). Resource substitution theory predicts that individual education is the main driver of health inequalities and parental education may either mitigate or amplify the effects of individual education (Ross & Mirowsky, 2011). Finally, social mobility theory indicates that the inter-generational movement across social strata, either upward or downward, is the main driver of inequalities (Hallqvist et al., 2004). Disentangling these alternative models with empirical data is relevant for public health, as each theory may inform specific actions to reduce educational inequalities in longevity, from the identification of groups at higher risk to the prioritization of family and/or institution related educational exposure.

Multi-generational educational trajectories have already been shown to affect all-cause mortality in different populations (Acacio-Claro et al., 2017; De Grande et al., 2015; Elo et al., 2014; Giesinger et al., 2014; Hayward & Gorman, 2004; Martikainen et al., 2020; Pudrovska & Anikputa, 2014; Wolfe et al., 2018). These studies were conducted in Finland, the United States, the United Kingdom and Belgium. Five studies (Belgium, Finland, USA) supported individual education as the main driver, while three supported that both parental and individual education affect longevity (Finland, United Kingdom, USA) (Elo et al., 2014; Giesinger et al., 2014; Martikainen et al., 2020). No study has yet examined the effect of intergenerational educational trajectories on longevity in other European countries.

Health inequalities have been observed to differ across country-related factors linked to social welfare (de Breij et al., 2020; Sieber et al., 2020). These macro-level factors are thought to impact health by moderating the effect of individual-level social determinants of health (Bambra, 2011). The most common operationalizations of these macro-determinants are based on welfare regimes, social policy institutions, and social expenditure (de Breij et al., 2020). While each approach has its own strengths and limitations, the first two can be particularly described as static, since they create broad non-changing clusters of countries. Also, they do not allow for comparison within their country clusters. The social expenditure approach involves a more dynamic country-by-country comparison, and is therefore the best suited to draw a cross-European comparison of inequalities in longevity.

It remains unclear whether country-level social expenditure modifies educational inequalities in longevity, potentially attenuating it. The empirical evidence in support of this hypothesis is still inconclusive in part due to contradicting findings across Europe (Brennenstuhl et al., 2012). For instance, while the Scandinavian welfare regime is widely regarded as the most generous in terms of social transfers, relative inequalities in mortality are higher there compared to Southern European states (Bambra, 2011; Mackenbach et al., 2008). At the same time, other research indicates that inequalities in self-reported health due to educational attainment are lower in countries with higher social expenditure (Álvarez-Gálvez & Jaime-Castillo, 2018). The effect of intergenerational educational trajectories on longevity may also vary cross-nationally, and this has not been examined yet. Thus, by using an intergenerational perspective on education we hope to shed more light on the contrasting findings from earlier research.

In this study, we aimed to utilize population-based multi-generational data to assess the role of parental and individual educational attainment in driving intergenerational inequalities in longevity among adults from 14 European countries. Further, we aimed to assess whether country-level social expenditure mitigates these inequalities.

## 2 Methods

### 2.1 Data source and study population

Our target population is adults born before 1965 and residing in European countries. The study population comprised community-dwelling adults participating in the Survey of Health, Ageing and Retirement in Europe (SHARE), a longitudinal cohort study across more than 20 European nations (Börsch-Supan et al., 2013, 2015; Börsch-Supan & Malter, 2015). The survey aims to examine the health of an ageing European population by collecting a multitude of socioeconomic, behavioral, and health-related data across the life course. Respondents are a representative sample of the whole population in each participating country. The SHARE study started in 2004 and has been conducted biennially, with a total of 8 waves until 2019/2020. Our study baseline was wave 5 (2013), since that was the first wave including an assessment of parental education. At that survey year there were 15 participating countries: Austria, Belgium, the Czech Republic, Denmark, Estonia, France, Germany, Israel, Italy, Luxembourg, the Netherlands, Slovenia, Spain, Sweden, and Switzerland.

**Fig. 1:**
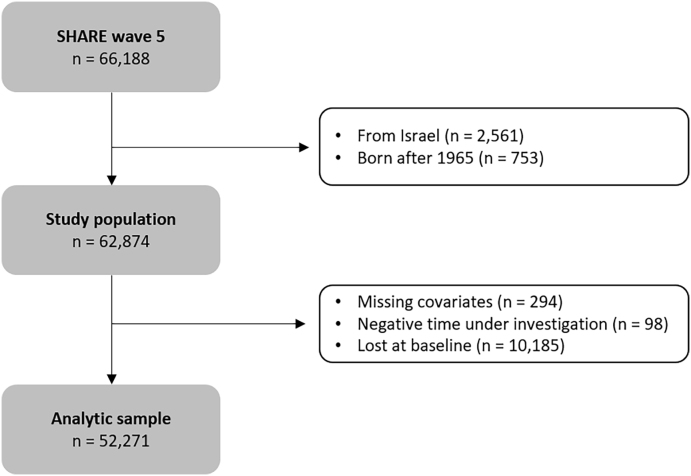
Flow chart of participants selection.

The study population, i.e. total number of respondents, was 66,188. The analytic sample included 52,271 participants, corresponding to 83% of the study population, as we excluded participants from Israel (n = 2561; Israel is not part of the European continent), born after 1965 (n = 535), with a negative time under investigation due to an incorrectly reported date of death (n = 98), with missing multimorbidity status or limitations with activities of daily living (n = 294), and lost at baseline (n = 10,429) (Fig. 1).

### 2.2 Causal model, exposure, outcome, and covariates

**Fig. 2:**
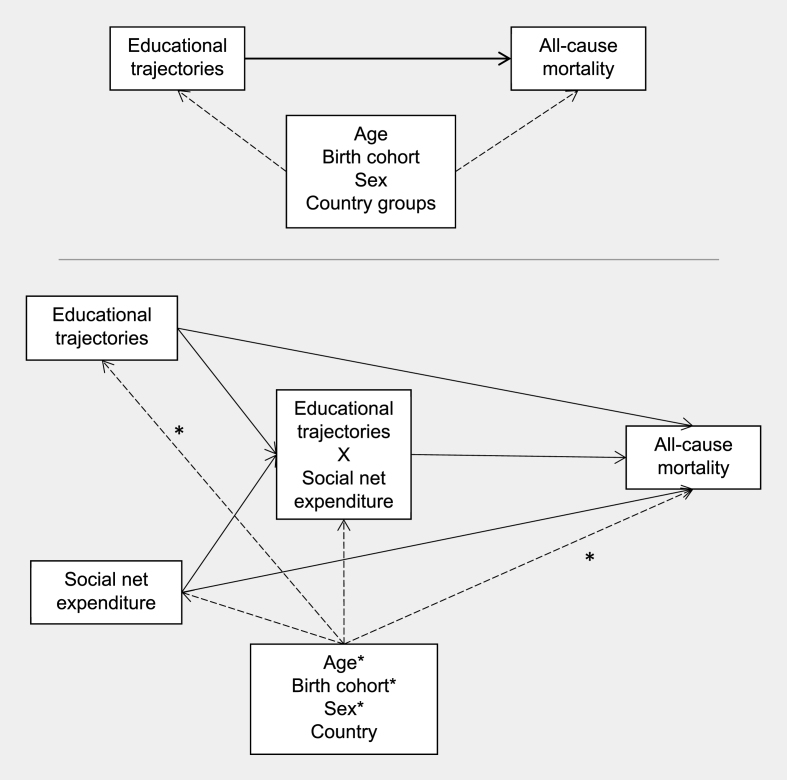
Directed acyclic graph (DAG) underlying our study, split by aim one (top) and aim two (bottom). Normal arrow: putative effect of interest. Dashed arrow: confounding factors. Confounding factors with asterisks only apply to the arrows with asterisks; “country” applies to all confounding arrows.

The causal model underlying our study is represented in Fig. 2. We designed it to focus on inequalities in all-cause mortality (outcome) driven by intergenerational educational trajectories (exposure) and modified by social net expenditure. In the model, the inequalities are represented by the total effect of the exposure on the outcome.

The design of the directed acyclic graph (DAG) for our second aim (bottom of Fig. 2) follows the recommendations of Attia et al. (2022) on how to represent effect modifications via DAGs. Here, the “Educational trajectories X Social net expenditure” node represents an additional effect on all-cause mortality due to the interaction between educational trajectories and social net expenditure.

Educational trajectories were constructed through the combination of individual and parental education. Both were self-reported by study participants. Parental education was operationalized as the highest achieved education between mother and father; in case one was missing, the other’s educational attainment was used. Both individual and parental educational attainment were categorized as “Low” for any achieved degree up to lower secondary education (International Standard Classification of Education (ISCED) 1997 levels 0–2) and “High” for upper secondary education and above (ISCED-1997 level 3 or above). Using this categorization, we obtained four educational trajectories: High-High, Low-High, High-Low, and Low-Low, where the first part denotes the parents’ education level and the second part the individual’s education level. This categorization follows previous research as well as documentation by Eurostat, where we combined “medium” (ISCED-1997 levels 3 and 4) and “high” (ISCED 1997 levels 5 and 6) education into a singular “High” category of educational attainment (Eurostat, 2011). In a sensitivity analysis, we also categorized individual educational attainment into “Low” (ISCED-1997 levels 0 and 1), “Medium” (ISCED-1997 levels 2 and 3), and “High” (ISCED-1997 levels 4 and above), to ascertain whether increased social expenditure may be more beneficial for the middle educated. This analysis focused on individual education only given the trade-off between the complexity of numerous trajectories and the available sample size.

Mortality data were collected during end-of-life surveys conducted every two years as part of the regular SHARE waves. In the case of death of a participant, proxies, e.g. family members or partners, were invited to participate and provided information on the date (month and year) and cause of death of the participant. For this study, we considered deaths due to all causes occurring at any time during follow-up, that is between wave 5 (2013) and wave 8 (2019/20).

Potential measured confounders were participant’s age at baseline, birth cohort (1909–1938, 1939–1945, 1946–1964), sex, and country of residence. These variables were self-reported and retrieved from the SHARE wave 5 baseline questionnaire. For analyses pertaining to our first aim, countries were categorized in four groups: Scandinavian countries (Sweden, Denmark), Central European countries (Austria, Germany, Netherlands, France, Switzerland, Belgium, Luxembourg), Southern European countries (Spain, Italy), and Eastern European countries (Czech Republic, Slovenia, Estonia). Countries were combined to reduce the risk of positivity violations. The choice of groups followed previous research describing differing intergenerational educational mobility patterns and mortality rates across these groups of countries (Schuck & Steiber, 2018; Torul & Oztunali, 2017).

Social expenditure was considered a country-level potential effect modifier and exposure (bottom of Fig. 2). Data on social expenditure came from the statistical office of the European Union Eurostat (Eurostat, 2022). Social expenditure was defined as public spending on social protection against risks such as unemployment, homelessness, sickness, or disability. We computed the net social protection as the percentage of social expenditure with respect to a country’s gross domestic product (GDP); Eurostat data is available from 2007 until 2018, and the net expenditure was approximately constant across this period. We considered the average social net protection spending between 2013 and 2018.

### 2.3 Statistical and sensitivity analyses

#### 2.3.1 Assessment of intergenerational inequalities in longevity and role of parental/individual education

We examined estimates of three effects comparing the intergenerational trajectories High-Low, Low-High, and Low-Low with the High-High trajectory, since we hypothesized participants in this group to have the lowest mortality rates. By doing so, we are able to disentangle among the three competing social theories and identify the role of parental and individual education in driving inequalities (Howe et al., 2016). We considered the following scenarios:

1. Similar effect estimates between the three trajectories indicate that both parental and individual education drive inequalities (cumulative advantage).
2. Similar effect estimates of having High-Low and Low-Low while the effect of having Low-High is negligible, indicate that individual education is the main driver (resource substitution).
3. Similar effect estimates of having Low-High and/or High-Low while Low-Low is negligible indicates that change, i.e. upward or downward social mobility, is the main driver (social mobility).

The internal validity of the effect estimates relies on the assumptions of positivity, consistency, no residual confounding, no measurement error of exposure/outcome/confounders, and correct specification of the statistical estimation model (Westreich, 2019).

Effects were measured as years of life lost (YLL) between ages 50 and 90. Years of life lost were calculated as differences of life expectancies between ages 50 and 90. We chose YLL as our effect measurement because it is based on years of life expectancy, therefore more directly related to longevity compared to traditional hazard differences or ratios. Further, it is a measure of inequalities on the absolute scale, thus more relevant in the evaluation of potential policy and public health actions on the examined exposure. Life expectancy for each level of exposure was computed as the area under the corresponding survival curve. The survival probability due to a certain educational trajectory was estimated as the predicted proportion of survivors based on the counterfactual scenario of every study participants having that educational trajectory and not being lost during follow-up. In practice, survival probabilities were estimated via the weighted Kaplan-Meier survival with age as time-scale.

Weights were the product of two separate stabilized inverse probability weights (IPWs) to account for (1) measured confounders and (2) potential non-random loss during follow-up (Cole & Hernán, 2008; Westreich, 2019). The IPW model for confounding included sex, birth period, and country groups. Age was indirectly adjusted for by using it as the scale of the time-to-event analysis. The IPW model for follow-up losses included sex, country, educational trajectory, birth period, baseline multimorbidity (min. 2 chronic diseases, self-reported), and limitations with activities of daily living (no limitations, 1 limitation, >2 limitations, self-reported). Finally, we ran sensitivity analyses to assess (1) the potential effect modification of sex and country groups and (2) the potential violation of the assumption of no IPWs model misspecification by incrementally truncating weights (Cole & Hernán, 2008).

Confidence intervals (CI) were generated via percentiles of 1000 bootstrap draws with replacement. Within each bootstrapped sample, the effect estimates were the average of 30 multiply imputed data sets for parental or individual education (n = 9278; 18%). We performed data imputations via chained equations under the hypothesis of missingness at random. The prediction variables in the imputation model were sex, multimorbidity, alcohol consumption (classified as high, moderate, and abstainer), limitations with activities of daily living, birth period, country, age at baseline and end of follow-up, censored age, and the cumulative hazard. Imputations were implemented with the mice R package (Van Buuren et al., 2019).

#### 2.3.2 Assessment of effect modification by social net expenditure

To assess variation of YLL by country-level social expenditure, we first estimated YLL per country, then we ran a meta regression with social net expenditure as explanatory variable (Harrer et al., 2021). Southern European countries (Italy and Spain) were excluded due to potential violation of the positivity assumption when estimating YLL for these countries; thus, we compared twelve out of the available fourteen countries. We repeated this analysis for life expectancies.

All analyses were run in R 4.1.2.

## 3 Results

### 3.1 Analytic sample characteristics

Characteristics of the analytic sample are reported in Table 1. Participants had a mean age of 67 years at baseline and were slightly more female (56%) than male. More than half were born after 1945, while approximately 20% were born during the Second World War (1939–1945). Southern countries had fewer participants with High-High (2%) and High-Low (3%) trajectories compared to other countries. Over approximately 7 years of follow-up, 6044 deaths occurred. The crude death rate was 2317 deaths per 100,000 person-years. Participants with High-High and Low-Low trajectories accounted altogether for 52% of the sample, that is more than half of the participants attained the same educational level as their parents. Approximately 25% of the participants experienced upward mobility and 5% downward mobility. Additionally, a high education was achieved by nearly eight out of ten participants with high educated parents, and by four out of ten participants with low educated parents. The remaining 18% of participants have missing information.

**Table 1:** Characteristics of analytic sample by educational trajectories, e.g. High-High = high parental education – high individual education. SD = standard deviation.

Characteristics	n (% or SD)	High-High	Low-High	High-Low	Low-Low
Number of participants	52,271	10,841 (21%)	13,118 (25%)	2523 (5%)	16,439 (31%)
**Sex**					
Female	29,159 (56%)	5798 (54%)	6704 (51%)	1647 (65%)	9701 (59%)
Male	23,112 (44%)	5043 (47%)	6414 (49%)	876 (35%)	6738 (41%)
**Age at baseline (years)**, mean and SD	67.2 (±10.0)	64.8 (±9.1)	65.7 (±9.3)	67.8 (±10.1)	70.5 (±10.4)
**Birth cohorts**					
1909–1938	12,850 (25%)	1736 (16%)	2493 (19%)	657 (26%)	6092 (37%)
1939–1945	10,890 (21%)	2202 (20%)	2635 (20%)	543 (22%)	3610 (22%)
1946–1964	28,531 (55%)	6903 (64%)	7990 (61%)	1323 (52%)	6737 (41%)
**Multimorbidity** (min. 2 chronic conditions)					
Yes	25,448 (49%)	4830 (45%)	5800 (44%)	1463 (58%)	9139 (56%)
No	26,823 (51%)	6011 (55%)	7318 (56%)	1060 (42%)	7300 (44%)
**Limitations with activities of daily living**					
No limitations	46,069 (88%)	9942 (92%)	11,898 (91%)	2156 (86%)	13,757 (84%)
1 limitation	3049 (6%)	525 (5%)	673 (5%)	179 (7%)	1167 (7%)
>2 limitations	3153 (6%)	374 (3%)	547 (4%)	188 (8%)	1515 (9%)
**Alcohol consumption**					
High	14,683 (28%)	3350 (31%)	3875 (30%)	578 (23%)	3923 (24%)
Moderate	21,478 (41%)	5346 (49%)	5961 (45%)	1067 (42%)	4952 (30%)
Abstainer	16,110 (31%)	2145 (20%)	3282 (25%)	878 (35%)	7564 (46%)
**Number of deaths** (2013–2020)	6044 (12%)	881 (8%)	1174 (9%)	332 (13%)	2752 (17%)
**Death rate**, crude (deaths per 100,000 person-years)	2317	1566	1755	2660	3548
**Country groups**					
Scandinavia	7385 (14%)	1605 (15%)	2483 (19%)	257 (10%)	1536 (9%)
Central Europe	22,150 (42%)	5721 (53%)	5621 (43%)	957 (38%)	4966 (30%)
Eastern Europe	12,741 (24%)	3330 (31%)	3212 (25%)	1240 (49%)	2653 (16%)
Southern Europe	9995 (19%)	185 (2%)	1802 (14%)	69 (3%)	7284 (44%)

Participants with a High-Low and Low-Low trajectory had a higher proportion of multimorbidity (58% and 56%) compared to participants with a Low-High and High-High trajectory (44% and 45%, respectively). The same pattern was observed for limitations with activities of daily living and number of deaths (17% versus 8%).

### *3.2* Intergenerational educational inequalities in longevity

**Fig. 3:**
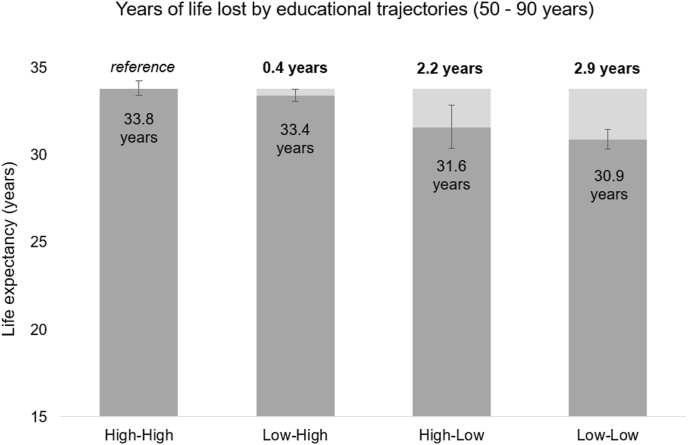
Life expectancy between ages 50 and 90 years and years of life lost (bold) due to different educational trajectories (parental-individual education). Standardized by age at baseline, sex, birth period, country group.

Life expectancies and years of life lost between ages 50 and 90 are reported in Table 2. The life expectancy associated with a High-High trajectory was 33.8 years (95% CI: 33.3 to 34.2). Compared to having a High-High educational trajectory, Low-High led to 0.4 (95% CI: 0.2 to 0.9) YLL, High-Low to 2.2 (95% CI: 1.0 to 3.5) YLL, and Low-Low to 2.9 (95% CI: 2.2 to 3.6) YLL (Table 2 and Fig. 3). To assess whether the YLL associated with High-Low and Low-Low were different, we directly compared the life expectancies related to these two exposure levels. The Low-Low vs High-Low estimate resulted in 0.7 (95% CI: 0.6 to 2.0) YLL, which was inconclusive given the wide compatibility interval.

**Table 2:** Life expectancy (years) between ages 50–90 years and years of life lost (YLL) due to different educational trajectories with respect to High-High (parental-individual education). Standardized by age at baseline, sex, birth period, country group.

Educational trajectory	Life expectancy (95% CI)	YLL (95% CI)	
High-High	**33.8** (33.3 to 34.2)		–
Low-High	**33.4** (33.1 to 33.7)	**0.4** (−0.2 to 0.9)	
High-Low	**31.6** (30.3 to 32.8)	**2.2** (1.0 to 3.5)	
Low-Low	**30.9** (30.3 to 31.4)	**2.9** (2.2 to 3.6)

There were 78 participants from Southern countries with a high value of the weights (>10), signaling a potential violation of the positivity assumption. To assess this, we ran additional analyses by implementing different model specifications for the weights related to measured confounding and by removing the Southern European countries (Table S1). Inequalities in these additional analyses were similar to those in main analyses, indicating the main results are robust to potential violations of the positivity assumption.

### 3.3 Country-level relationship between YLL and social net expenditure

The per-country social net expenditures as percentage of GDP were: 30% for France, 27% for Denmark, 26% for Belgium and Austria and Germany, 25% for Sweden and Italy, 23% for Spain and Slovenia, 22% for Netherlands, 21% for Switzerland, 19% for Luxembourg, 18% for the Czech Republic, and 15% for Estonia.

A higher social net expenditure was associated with a longer life expectancy but not smaller YLL. Specifically, a 1% increase in social net expenditure was associated with additional 0.2 years of life expectancies for High-Low (95% CI: 0 to 0.5), and Low-Low (95% CI: 0 to 0.5) (Table 3 and Fig. S1). For the other two trajectories, the estimates were inconclusive as they were less precise, namely 0.2 (95% CI: 0.2 to 0.6) years and 0.2 (95% CI: 0.4 to 0.8) years for High-High and Low-High, respectively. Finally, a 1% increase in social net expenditure was associated with negligible changes in YLL for all levels of exposure (Table 3).

**Table 3:** Country-level relationship between life expectancy between 50 and 90 years of age with social net expenditure (mean percentage of GDP between 2013 and 2018). LE: life expectancy between age 50 and 90; YLL years of life lost between age 50 and 90 with respect to reference educational trajectory High-High (parental-individual education). LE and YLL were standardized by age at baseline, sex, and birth period.

Educational trajectory	Change in LE per 1% increase in social expenditure (95% CI)	Change in YLL per 1% increase in social expenditure (95% CI)
High-High	**0.2** (−0.2 to 0.6)	**-**
Low-High	**0.2** (−0.4 to 0.8)	**0.01** (−0.3 to 0.3)
High-Low	**0.2** (0 to 0.5)	**0.007** (−0.1 to 0.2)
Low-Low	**0.2** (0 to 0.5)	**−0.02** (−0.2 to 0.1)

### 3.4 Sensitivity analyses

Years of life lost due to intergenerational educational trajectories were larger in men compared to women (Table 4). For men, parental education appeared to moderate intergenerational inequalities driven by low individual education, as having High-Low led to smaller YLL (2.7, 95% CI: 1.4 to 5.1) than having Low-Low (4.0, 95% CI: 2.6 to 4.6). This moderation was not observed for women: High-Low led to 1.7 (95% CI: 1.1 to 3.3) YLL while Low-Low led to 2.0 (95% CI: 1.3 to 2.7) YLL.

**Table 4:** Years of life lost (YLL) between ages 50–90 years due to the effect of educational trajectories, stratified by country groups and by sex. Reference = High-High (parental-individual education). Standardized by age at baseline, sex, birth period, country groups.

	Low-High vs. High-High	High-Low vs. High-High	Low-Low vs. High-High
	**YLL** (95% CI)	**YLL** (95% CI)	**YLL** (95% CI)
**Total**	**0.4** (−0.2 to 0.9)	**2.2** (1.0 to 3.5)	**2.9** (2.2 to 3.6)
**Sex**			
Female (n = 29,159)	**0.2** (−0.4 to 0.7)	**1.7** (1.1 to 3.3)	**2.0** (1.3 to 2.7)
Male (n = 23,112)	**0.8** (−0.6 to 1.2)	**2.7** (1.4 to 5.1)	**4.0** (2.6 to 4.6)
**Country**			
Central Europe (n = 22,150)	**0.1** (−0.7 to 0.5)	**2.7** (0.8 to 4.9)	**2.9** (1.9 to 4.0)
Eastern Europe (n = 12,741)	**0.5** (−0.4 to 1.4)	**2.3** (0.8 to 3.9)	**3.7** (2.3 to 5.0)
Scandinavia (n = 7385)	**0.6** (−1.6 to 0.3)	**2.6** (−0.6 to 5.3)	**1.5** (−0.3 to 3.8)
Southern Europe (n = 9995)	**1.7** (−1.0 to 4.6)	**0.2** (−5.2 to 6.6)	**2.6** (−0.2 to 5.3)

Pattern of years of life lost varied slightly across country groups (Table 4). Inequalities for Central Europe and Scandinavia were in line with the main analysis. Effect estimates for Eastern Europe indicated that low individual education drove intergenerational inequalities while parental education moderated them. Specifically, having a high parental education mitigated the inequalities, as having High-Low led to smaller YLL than having Low-Low. Finally, estimates for Southern European countries were inconclusive as their precision was low.

Participants lost at baseline (n = 10,429; 20%) were comparable to the analytic sample in their baseline characteristics (Table S2). This indicates findings from the analytic sample can be generalized to the study population. Similarly, when truncating weights, inequalities were similar to those reported in main analyses (Table S3 and Table S4), suggesting negligible bias from the potential miss-specification of the IPWs models.

When categorizing individual education in three levels – low (27%), medium (51%), and high (21%) – for 1% increase in social net expenditure we observed an increase in life expectancy by 0.2 years (95% CI: 0 to 0.4) when having high education, 0.3 years (95% CI: 0 to 0.5) when having medium education, and 0.2 years (95% CI: 0.3 to 0.8) when having low education (Fig. S2). Finally, a 1% increase in social net expenditure was associated with negligible changes in YLL: 0.02 years (95% CI: 0.2 to 0.1) for medium education and 0.05 years (95% CI: 0.5 to 0.6) for low education. Overall, this indicates that our main findings are robust with respect to a different operationalization of the exposure.

## 4 Discussion

We assessed inequalities in longevity due to intergenerational educational trajectories among older adults from 14 European countries. There were approximately 2.5 years of life lost when having a low individual education regardless of parental education, indicating that inequalities were driven by individual education and that the resource substitution model held. To place the approximately 2.5 years of life lost into context, one multicohort study in seven high-income WHO member states estimated the years of life lost between ages 40 and 85 years to be around 0.5 years for high alcohol intake, 0.7 years for obesity, 1.6 years for hypertension, 2.4 years for physical inactivity, 3.9 years for diabetes, and 4.8 years for current smoking (Stringhini et al., 2017). Additionally, we assessed the potential mitigation of inequalities by increased social expenditure of the country of residence, and observed that the higher the social expenditure, the larger the life expectancy, but not smaller the inequalities.

Our work expands previous studies in European populations about the role of parent and offspring educations in shaping intergenerational inequalities in longevity for cohorts born before 1965. In Finnish birth cohorts from 1935 to 1971, childhood social conditions were observed to be a significant predictor of adult mortality, but adulthood socioeconomic conditions explained most of this association. This indicated that individual socioeconomic conditions during adulthood were the main drivers of inequalities in mortality (Elo et al., 2014; Martikainen et al., 2020). Conversely, in a study on British participants born in 1946, investigators observed that both early childhood and adulthood socioeconomic conditions contributed to inequalities in all-cause premature mortality (Giesinger et al., 2014). Our study adds to these findings by assessing them on a larger scale, i.e. across 14 other European countries, and by observing that individual education was the main driver of intergenerational inequalities in longevity.

Our results are in line with the resource substitution hypothesis of health and education. This theory states that higher parental education provides resources that increase the likelihood of their offspring also achieving a higher education, thus decreasing their mortality risk. The effect of parental education on longevity therefore seems to be indirect rather than direct (Ross & Mirowsky, 2011). Indeed, in our analytic population the likelihood of achieving a high education was twofold in individuals having high educated parents compared to those having low educated parents. Further, we observed a moderating effect of parental education for men and in Eastern Europe. Notably, a high parental education mitigated intergenerational inequalities driven by low individual education. In summary, it can be said that while individual education appears to be the main driver of inequalities in longevity, it functions within the context of parental education and should be understood as part of a larger web of socioeconomic determinants of health.

Increased social expenditure was associated with an increase in life expectancy, similar to what previous studies in multiple countries have reported (Bergqvist et al., 2013; Bradley et al., 2011). This finding may underlie the protection of high social expenditure from the adverse health effects of poverty, by allowing people to invest in human capital such as education (Reynolds & Avendano, 2018), and reducing exposure to health risk factors, such as chronic stress which has been linked to various cardio-metabolic disorders (Kivimäki et al., 2022). Social expenditure is theorized to strengthen human agency and support peoples’ capacities to deal with stressful life events (Dahl & van der Wel, 2013). One study across 30 OECD countries, including non-European countries such as Japan, Mexico, or the United States, assessed the association of social expenditure with individual-level years of life lost between birth and age 69 and reported a positive effect of increased spending (Bradley et al., 2011). The authors conclude that social spending has beneficial effects on population health, beyond that of health spending alone.

Increased social net expenditure was not associated with reduced inequalities in longevity. This finding may have multiple explanations. First, only specific social expenditures, e.g. related to healthcare, might mitigate inequalities, as reported in at least one study (Vavken et al., 2012). As such, the use of total social expenditure may have blurred the examined relationship. Second, we were unable to assess social expenditure across the whole life course and therefore could not account for the fact that people benefit most from welfare at different life periods, e.g. educational expenditure before adulthood and pension benefits in older life (de Graaf & Maier, 2017).

Our study has limitations. Firstly, there is potential bias in both the exposure and outcome of interest. For the exposure, our findings may be subject to misclassification bias since education is self-reported. For educational attainment, the comparable results from classifying individual education into three instead of two levels indicate that the bias due to misclassification is likely to be small. Further, a study investigating the quality of retrospective childhood information provided in SHARE concluded that generally, respondents remembered their childhood living conditions with high accuracy (Havari & Mazzonna, 2015). For the outcome, mortality data is self-reported via proxies of the deceased, thus errors in the exact dates of death are possible. However, the ensuing bias is likely small (days) compared to the size of our estimated effects (years).

Secondly, residual confounding can be present. As this is an observational study, we may have unmeasured confounding, and we could not adjust for additional measured potential confounders due to positivity restriction. However, we note that the observed size of educational inequalities in our study are consistent with social inequalities observed in another study of larger sample size and with different cohorts (Stringhini et al., 2017).

Third, our findings may not be generalizable to the target population. There could be selection bias as our study population comprised individuals that survived until approximately age 50. This may have resulted in an underestimation of the inequalities (Mayeda et al., 2018). Finally, there might be potential violations of the consistency assumption due to the chosen exposure. We measured education as the highest attained degree without capturing duration or quality of education, both of which may be differentially associated with mortality (Rehkopf et al., 2016). Thus, there remains a gap between our findings and specific targets for potential interventions.

One key strength of our study is the utilization of a population-based multi-generational and multi-country data sample. Further, we adopted a causal framework with transparent identifying assumptions to estimate marginal inequalities, rather than conditional hazard ratios as has been done in previous studies. The latter ones are known to provide potentially biased effect estimates due to both non-collapsibility and implicit selection bias of the hazard ratio (Daniel et al., 2021; Hernán, 2010).

## 5 Conclusions

We provide empirical evidence that low individual education drives intergenerational inequalities in longevity among adult Europeans and that these inequalities were not mitigated through increased social net expenditure during their older life. Thus, our study supports the importance of achieving a high education, and of interventions facilitating it. One example would be the various policies implemented in Ireland to reduce financial barriers to achieving higher education, such as the “free education scheme” of 1967 or the removal of higher education tuition fees in 1996 (McCoy & Smyth, 2011; O’Donoghue et al., 2016). The need for better educational outcomes for European citizens has also been acknowledged by the European Commission in its updated council recommendation on school success, calling on all member states to strengthen their educational systems to reduce early school leaving (Union, 2022). From a country-level perspective, further research could build upon our findings to examine more specific policies of social spending that may be able to diminish inequalities in longevity. Particularly, our study highlights the need to examine the effect of welfare systems earlier in life to truly understand how welfare may impact longevity in the long run. This would require younger cohorts that are followed up for longer periods of time, but could create valuable information for public health specialists and policy makers aiming to diminish social inequalities in longevity.

## Funding

This project did not receive any specific funding.

## Data sharing

Data is available via registration to the SHARE project website (see www.share-project.org).

## Author contributions

Cornelia Wagner: Methodology, Formal analysis, Writing - Original Draft. Stéphane Cullati: Writing - Review & Editing. Stefan Sieber: Writing - Review & Editing. Tim Huijts: Writing - Review & Editing. Arnaud Chiolero: Writing - Review & Editing. Cristian Carmeli: Conceptualization, Methodology, Formal analysis, Writing - Review & Editing, Supervision.

## Declaration of competing interest

We declare no conflicts of interest.

## Data Availability

The authors do not have permission to share data.
