# Supplementary material for: Intergenerational educational trajectories and inequalities in longevity: A population-based study of adults born before 1965 in 14 European countries
Source: SSM Popul Health. 2023 Feb 22;22:101367. doi: 10.1016/j.ssmph.2023.101367 (PMC9974424; doi:10.1016/j.ssmph.2023.101367)

**Supplementary materials**

***Table S1.*** *Results of sensitivity analyses to assess potential violations of positivity in main analyses. Inequalities in longevity due to intergenerational trajectories are measured via years of life lost (YLL) with respect to the reference High-High trajectory. Analysis 1 (reported as main results) included country groups among the confounders the survival curves were standardized to and included participants from Southern Europe. Analysis 2 included country groups as a potential confounding factor, but excluded participants from Southern Europe. Analysis 3 did not include country groups into the confounders and included participants from Southern Europe. Analysis 4 excluded country groups from the confounders and excluded participants from Southern Europe.* *SE countries = Southern European countries. Reference = High-High (parental-individual education). Standardized by age at baseline, sex, birth period.*

| **Educational trajectory** | **Analysis 1** | **Analysis 2** | **Analysis 3** | **Analysis 4** |
| --- | --- | --- | --- | --- |
|  | **YLL** (95% CI) | **YLL** (95% CI) | **YLL** (95% CI) | **YLL** (95% CI) |
| **Low-High** vs High-High | **0.4** (-0.1 to 0.9) | **0.1** (-0.1 to 0.9) | **0.2** (-0.2 to 0.7) | **0.0** (-0.4 to 0.6) |
| **High-Low** vs High-High | **2.4** (1.1 to 3.6) | **2.2** (1.1 to 3.6) | **2.8** (1.7 to 3.9) | **3.0** (1.8 to 4.0) |
| **Low-Low** vs High-High | **2.9** (2.2 to 3.8) | **3.0** (2.2 to 3.8) | **2.7** (2.2 to 3.3) | **2.9** (2.1 to 3.7) |

***Table S2.*** *Sample characteristics for participants lost at baseline. Educational trajectories correspond to parental-individual educational attainments, e.g. High-High = high parental education – high individual education. SD = standard deviation.*

| **Characteristics** | **Analytic sample**  **n (% or SD)** | **Lost at baseline**  **n (% or SD)** |
| --- | --- | --- |
| Number of participants | 52,271 | 10,429 |
| **Sex** |  |  |
| Female | 29,159 (56%) | 5,672 (54%) |
| Male | 23,112 (44%) | 4,757 (46%) |
| **Age (years)**, mean and SD | 67.2 (± 10.0) | 65.9 (± 10.5) |
| **Birth cohorts** |  |  |
| 1909-1938 | 12,850 (25%) | 2,306 (22%) |
| 1939-1945 | 10,890 (21%) | 1,836 (18%) |
| 1946-1964 | 28,531 (55%) | 6,287 (60%) |
| **Multimorbidity** (min. 2 chronic conditions) |  |  |
| Yes | 25,448 (49%) | 4,668 (45%) |
| No | 26,823 (51%) | 5,761 (55%) |
| **Limitations with activities of daily living** |  |  |
| No limitations | 46,069 (88%) | 9,290 (89%) |
| 1 limitation | 3,049 (6%) | 541 (5%) |
| Min. 2 limitations | 3,153 (6%) | 598 (6%) |
| **Number of deaths** (2013-2020) | 6,044 (12%) | - |
| **Educational trajectories** |  |  |
| High-High (reference) | 10,841 (21%) | 1,888 (18%) |
| Low-High | 13,118 (25%) | 2,683 (26%) |
| High-Low | 2,523 (5%) | 496 (5%) |
| Low-Low | 16,439 (31%) | 3,333 (32%) |
| Missing | 9,350 (18%) | 2,029 (20%) |

***Table S3.*** *Life expectancies (years) between ages 50 – 90 years and years of life lost (YLL) due to different educational trajectories when truncating IPWs to the 1^st^ and 99^th^ percentiles. Reference = High-High (parental-individual education). Standardized by age at baseline, sex, birth period, country group.*

| **Educational trajectory** | **Life expectancies** (95% CI) | **YLL** (95% CI) |
| --- | --- | --- |
| High-High | **33.9** (33.5 to 34.2) | **-** |
| Low-High | **33.4** (33.0 to 33.7) | **0.5** (0.1 to 0.9) |
| High-Low | **31.6** (30.4 to 32.6) | **2.2** (1.1 to 3.5) |
| Low-Low | **30.8** (30.3 to 31.4) | **3.0** (2.4 to 3.7) |

***Table S4.*** *Life expectancies (years) between ages 50 – 90 years and years of life lost (YLL) due to different educational trajectories when truncating IPWs to the 1^st^ and 95^th^ percentiles. Reference = High-High (parental-individual education). Standardized by age at baseline, sex, birth period, country group.*

| **Educational trajectory** | **Life expectancies** (95% CI) | **YLL** (95% CI) |
| --- | --- | --- |
| High-High | **33.9** (33.5 to 34.2) | **-** |
| Low-High | **33.4** (33.1 to 33.8) | **0.4** (0.0 to 0.9) |
| High-Low | **31.5** (30.4 to 32.6) | **2.3** (1.2 to 3.5) |
| Low-Low | **31.0** (30.5 to 31.5) | **2.8** (2.2 to 3.5) |

***Figure S1.*** *Correlation between life expectancy (years, ages 50-90 years) and social net expenditure as percentage of GDP (mean, 2013-2018) by country.* *Split by educational trajectories, e.g. High-High = high parental education – high individual education.* *Bubbles correspond to individual countries; sizes correspond to precision of the estimate, i.e. the larger the circle the wider the 95% confidence interval.*


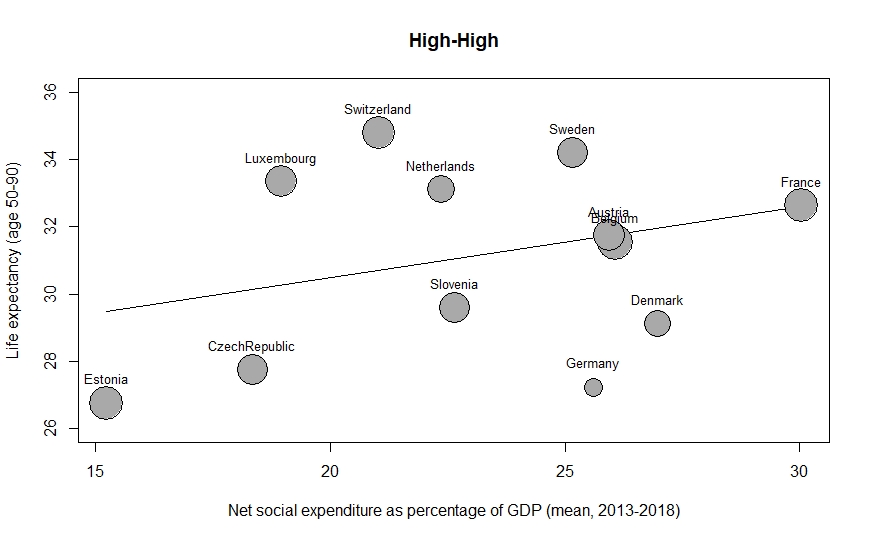

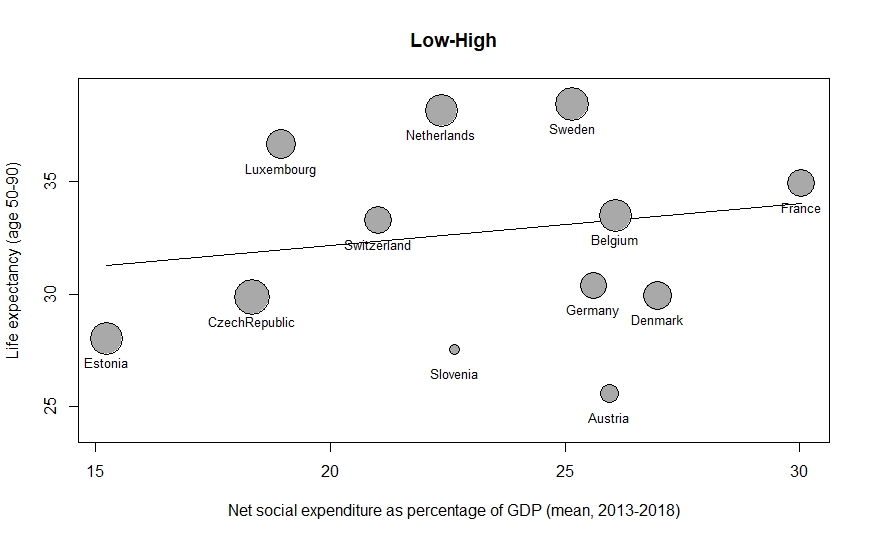

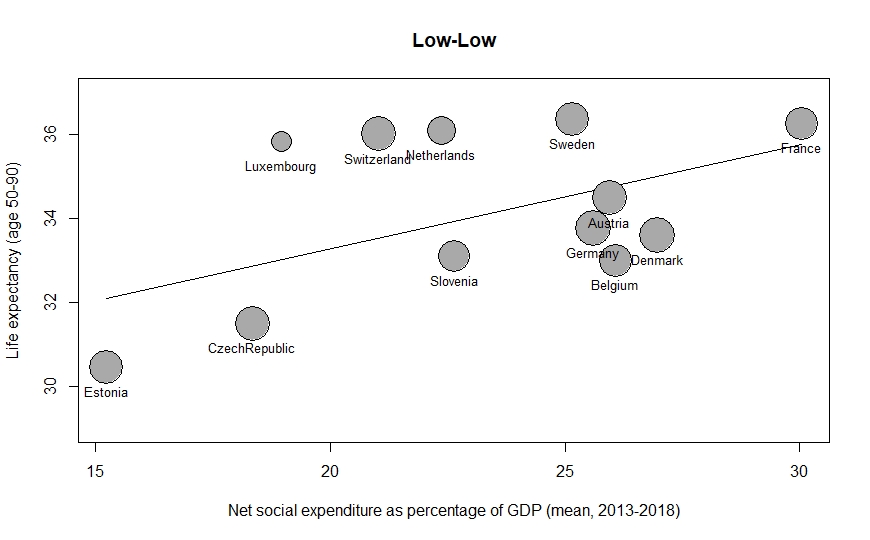

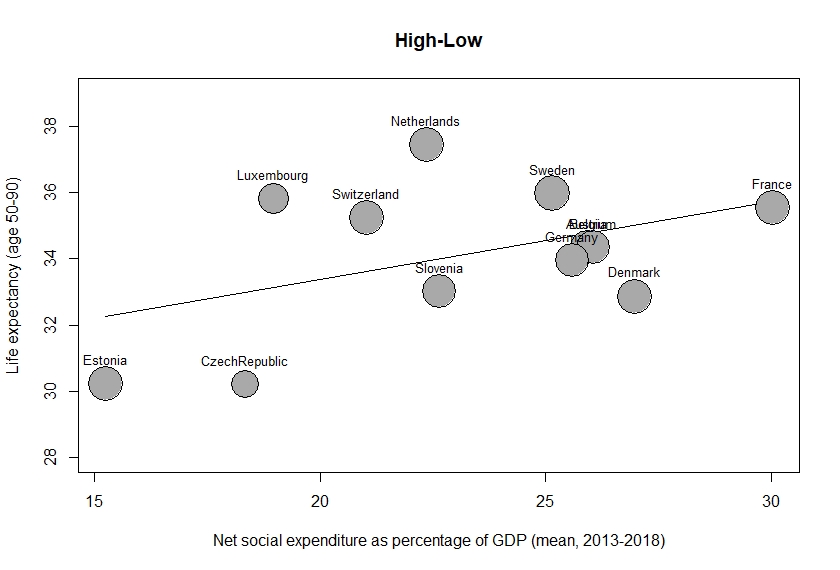


**Figure S2.** *Correlation between life expectancy (years, between 50-90 years of age) and social net expenditure as percentage of GDP (mean, 2013-2018) by country.* *Split by individual educational attainment.* *Bubbles correspond to individual countries; sizes correspond to precision of the estimate, i.e. the larger the circle the wider the 95% confidence interval.*
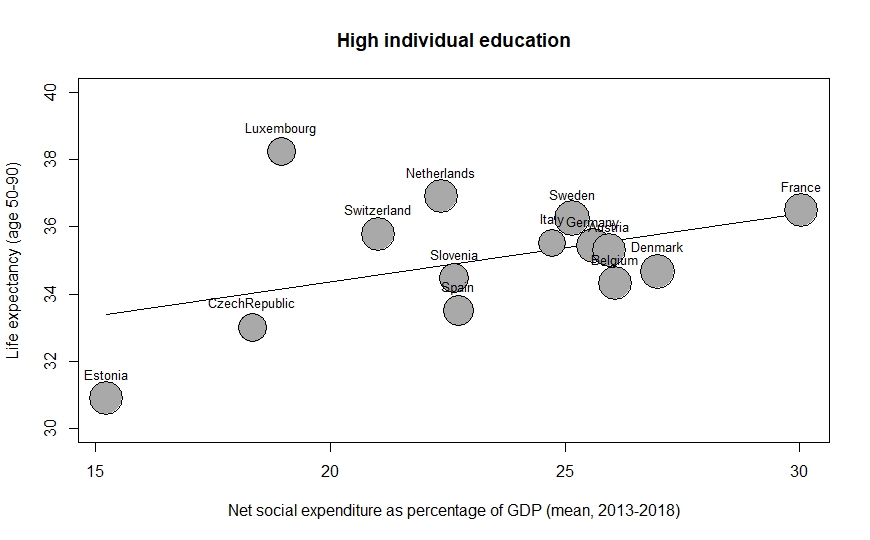


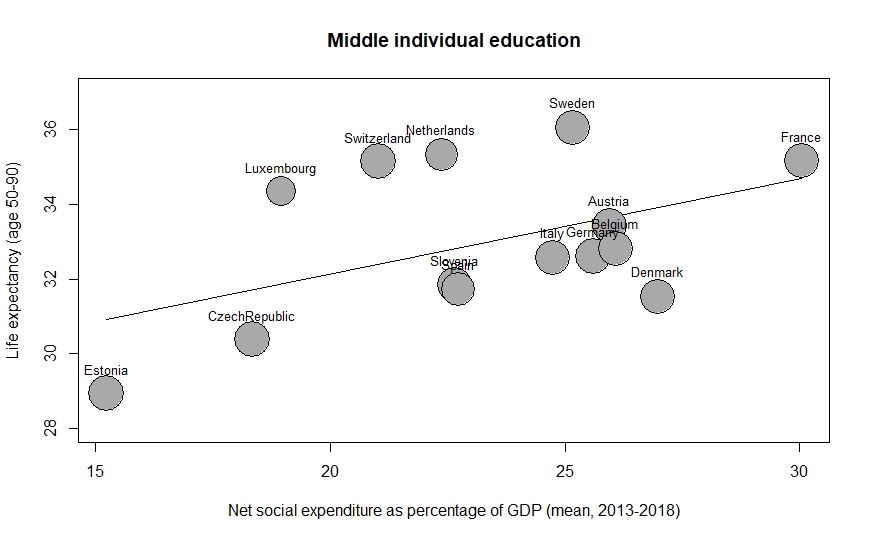


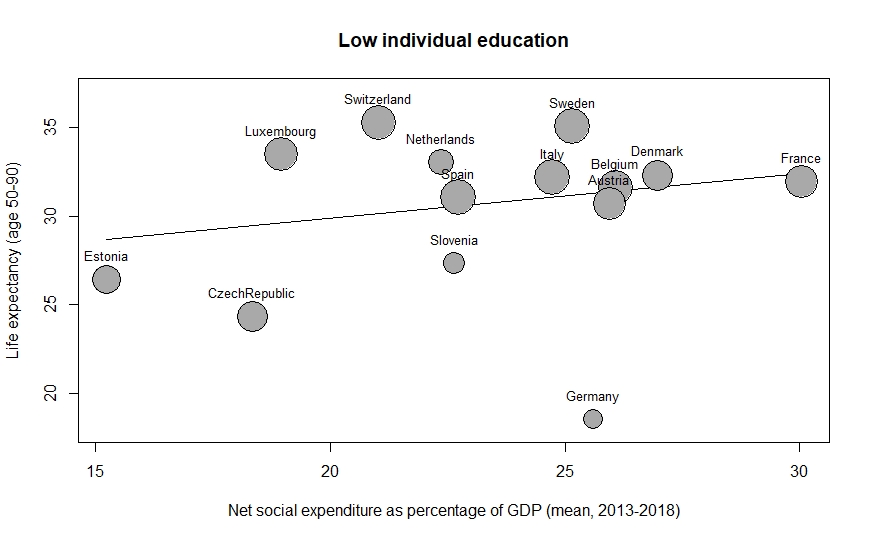

Supplement: Multimedia component 1 [file mmc1.docx]
